# Dissection of depression heterogeneity using proteomic clusters

**DOI:** 10.1017/S0033291721004888

**Published:** 2023-05

**Authors:** Marije van Haeringen, Yuri Milaneschi, Femke Lamers, Brenda W.J.H. Penninx, Rick Jansen

**Affiliations:** Department of Psychiatry, Amsterdam UMC, Vrije Universiteit, Amsterdam Public Health Research Institute and Amsterdam Neuroscience, Amsterdam, The Netherlands

**Keywords:** Depression, heterogeneity, proteomics, WGCNA

## Abstract

**Background:**

The search for relevant biomarkers of major depressive disorder (MDD) is challenged by heterogeneity; biological alterations may vary in patients expressing different symptom profiles. Moreover, most research considers a limited number of biomarkers, which may not be adequate for tagging complex network-level mechanisms. Here we studied clusters of proteins and examined their relation with MDD and individual depressive symptoms.

**Methods:**

The sample consisted of 1621 subjects from the Netherlands Study of Depression and Anxiety (NESDA). MDD diagnoses were based on DSM-IV criteria and the Inventory of Depressive Symptomatology questionnaire measured endorsement of 30 symptoms. Serum protein levels were detected using a multi-analyte platform (171 analytes, immunoassay, Myriad RBM DiscoveryMAP 250+). Proteomic clusters were computed using weighted correlation network analysis (WGCNA).

**Results:**

Six proteomic clusters were identified, of which one was nominally significantly associated with current MDD (*p* = 9.62E-03, Bonferroni adj. *p* = 0.057). This cluster contained 21 analytes and was enriched with pathways involved in inflammation and metabolism [including C-reactive protein (CRP), leptin and insulin]. At the individual symptom level, this proteomic cluster was associated with ten symptoms, among which were five atypical, energy-related symptoms. After correcting for several health and lifestyle covariates, hypersomnia, increased appetite, panic and weight gain remained significantly associated with the cluster.

**Conclusions:**

Our findings support the idea that alterations in a network of proteins involved in inflammatory and metabolic processes are present in MDD, but these alterations map predominantly to clinical symptoms reflecting an imbalance between energy intake and expenditure.

## Introduction

Major depressive disorder (MDD) is a leading cause of disability worldwide, with the number of people affected estimated at 322 million (Institute for Health Metrics and Evaluation, 2018). Treatment often includes antidepressant medication, although efficacy is mild to moderate (Murrough & Charney, 2012). Many studies concentrate on attempts to further unravel the molecular mechanisms underlying MDD in order to identify biomarkers that could be used as new targets for treatment. Previous large meta-analyses showed that MDD is associated with immunological and metabolic dysregulations as marked by increased levels of inflammatory markers (interleukin (IL)-6, C-reactive protein (CRP), tumor necrosis factor (TNF)-*α*) and metabolic markers (triglycerides, insulin sensitivity, adiposity) (Dowlati et al., 2010; Hiles, Lamers, Milaneschi, & Penninx, 2017; Howren, Lamkin, & Suls, 2009; Kan et al., 2013; Köhler et al., 2017; Pan et al., 2012; Smith, Au, Ollis, & Schmitz, 2018; Vancampfort et al., 2014), although meta-analytic estimates were of small effect size and characterized by high heterogeneity.

The association with biological dysregulations may vary as a function of depression heterogeneity; patients with the same MDD diagnosis endorse very different symptom profiles that, in turn, may be differentially related to underlying biological dysregulations. For example, MDD with anxious distress has been associated with increased immunological alterations, more cortical thinning, and corticolimbic dysfunctions as compared with depression without anxious distress (Gaspersz, Nawijn, Lamers, & Penninx, 2018). Also, recent evidence suggests that the link with adverse inflammatory and metabolic dysregulations seems stronger in patients reporting atypical depressive symptoms characterized by altered energy homeostasis, such as hypersomnia, increased appetite, weight gain, energy loss and leaden paralysis (Glaus et al., 2013; Lamers et al., 2020, 2013; Milaneschi et al., 2016; van Reedt Dortland, Giltay, van Veen, van Pelt, et al., 2010; van Reedt Dortland, Giltay, van Veen, Zitman, et al., 2010). Building on this evidence, a recent hypothesis postulates the existence of an ‘immuno-metabolic depression’ (IMD) dimension emerging from the clustering of atypical, energy-related clinical symptoms with inflammatory and metabolic dysregulations (Milaneschi, Lamers, Berk, & Penninx, 2020).

However, which symptoms best identify depression subtypes, and which biomarkers best tag the underlying mechanism, remains to be studied further. Furthermore, most previous research examining biological dysregulations in MDD and related individual symptoms considered only a single or few biomarkers, which may not be sufficient to capture the complexity of biological mechanisms. We hypothesize that networks of biomarkers may better capture complex interactions underlying biological alterations than single biomarkers.

We performed cluster analysis using weighted correlation network analysis (WGCNA) (Langfelder & Horvath, 2008), which has been applied in the past to gene expression (Jansen et al., 2016) and proteomic data (Zhang et al., 2018) to reveal network-level mechanisms (Vella, Zoppis, Mauri, Mauri, & Di Silvestre, 2017). For example, *MacDonald and colleagues* used WGCNA to identify alterations in the expression of glutamate signaling pathway proteins in schizophrenia patients (MacDonald et al., 2015). In the present study, we used a proteomic assay with 171 markers that quantifies serum proteins involved in hormonal, immunological and metabolic pathways. We investigate which clusters of these proteins are most strongly associated to MDD and which depressive symptoms are major drivers of these associations.

## Methods

### Sample description

The data originated from the Netherlands Study of Depression and Anxiety (NESDA), a longitudinal cohort study on factors that influence the development and course of anxiety and depressive disorders (Penninx et al., 2008). The baseline sample of the NESDA cohort consists of 2981 participants (age 18–65) with and without (a history of) depressive or anxiety disorders. The subjects were recruited from the general population (*N* = 564), primary care (*N* = 1610) and specialized mental health care (*N* = 807). All participants completed the baseline assessment, consisting of a face-to-face interview, written questionnaires and biological measurements. The research protocol was approved by the Ethical Committee of the participating centers, and all subjects provided written informed consent. Proteomic analytes were determined in the subset of NESDA participants who participated in both baseline and 2-year follow-up assessments and for whom sufficient serum (~1 ml) from the baseline assessment was available (*N* = 1837). This data has been used before to study associations with depression (Bot et al., 2015) and depression subtypes (Lamers et al., 2016).

### Major depressive disorder and depressive symptoms

The presence of depressive disorders (MDD and dysthymia) and anxiety disorders (panic disorder, social phobia, generalized anxiety disorder and/or agoraphobia) was established using the Composite Interview Diagnostic Instrument (CIDI, version 2.1, World Health Organization, 1997). For this study, we removed participants with lifetime anxiety disorder and lifetime dysthymia from the healthy controls. This lead to a selection of 1621 samples: healthy controls (no depressive or anxiety disorder in a lifetime, *N* = 426), remitted MDD (lifetime MDD, but not in past 6 months, *N* = 483) and current MDD (MDD in past 6 months, *N* = 712). The severity of specific depressive symptoms in the week before the baseline interview was measured using the 30-item self-report Inventory of Depressive Symptomatology (IDS-SR_30_), which includes core symptoms of MDD, melancholic and atypical features, and other commonly associated symptoms (Rush, Gullion, Basco, Jarrett, & Trivedi, 1996). The specific depressive symptoms and a measure for the overall depression severity (total IDS score) – defined as the sum of the score of all items – were derived from the IDS. To study the individual symptoms, the four answer categories of the IDS-SR_30_ were dichotomized, that is, levels 0 and 1 code for low and levels 2 and 3 for high symptom endorsement, as has been done before (Khan et al., 2006). For each symptom, a 4-level factor was created: (1) controls, (2) remitted MDD, (3) current MDD with low symptom endorsement and (4) current MDD with high symptom endorsement. The five energy-related symptoms as identified with the IDS questionnaire are increased appetite, increased weight, energy loss, leaden paralysis and hypersomnia (Lamers et al., 2020; Milaneschi et al., 2020)

### Proteomic analytes

Protein measures have been described in detail previously (Bot et al., 2015; Lamers et al., 2016). In short, after an overnight fast, blood samples were taken in five research sites (Amsterdam, Leiden, Groningen, Emmen and Heerenveen), and stored at −80°C. The samples were shipped on dry ice to a Clinical Laboratory Improvement Amendments-certified laboratory (Myriad RBM; Austin, TX, USA). A panel of 243 analytes (Myriad RBM DiscoveryMAP 250+) involved in various hormonal, immunological, metabolic and neurotrophic pathways were assessed in serum using multiplexed microbead immunoassays. Three duplicate quality controls with different protein concentrations were included for each batch. Average inter- and intra-assay variability were respectively 10.6% (range 5.5–32.5%) and 5.6% (range 2.5–15.8%). Proteomic analytes with more than 30% missing data were excluded from the analysis, leaving 171 analytes for current analyses (online Supplementary Table S1).

### Covariates

Covariates were a priori selected based on previous research and include batch number (total of 26 batches), research site, sex, age, years of education and various somatic and lifestyle variables: number of self-reported chronic diseases under treatment, alcohol consumption (drinks/week), current smoking status (yes/no), physical activity and body mass index (BMI). Physical activity was measured with the International Physical Activity Questionnaire (Craig et al., 2003) and expressed as metabolic equivalent minutes per week (Lamers et al., 2016).

### Statistical methods

In line with previous studies (Bot et al., 2015; Lamers et al., 2016), missing values were imputed by the median value of the corresponding analyte. Values outside the range of detection were imputed with the upper or lower limit of detection. Prior to analysis, all analytes were quantile-normal transformed to stabilize the variance.

Clusters of correlated proteins were identified using correlation networks constructed with WGCNA (version 1.67) package in the R programming environment. A detailed description of the algorithm can be found elsewhere (Langfelder & Horvath, 2008). WGCNA co-expression networks were constructed using the unsigned similarity measure, where the connection strength between two proteins reflected the absolute value of their correlation regardless of positive or negative direction.

The Pearson correlation matrix was raised to the fourth power to meet the scale-free topology criterium. Clusters of analytes were detected using hierarchical clustering, where a dendrogram is constructed with branches corresponding to the clusters. The minimum number of analytes per cluster was set to five. The other settings remained at default values. The eigenprotein, representing the weighted average level of the corresponding cluster, was calculated for each of the identified clusters, defined as the first principal component of the cluster. Furthermore, the importance of each analyte in a cluster was quantified by the ‘module membership’ as computed by WGCNA, which is defined as the correlation between the circulating levels of the individual proteins of the cluster and its eigenprotein. Next, the biological functions of the proteins contained in each of the clusters were investigated. We studied whether the identified clusters were enriched for categories from the Gene Ontology Biological Process (GO), Reactome, Kyoto Encyclopedia of Genes and Genomes (KEGG) and BioCarta databases using the over-representation analysis of ConsensusPathDB (FDR < 0.1) (Kamburov et al., 2011; Kamburov, Wierling, Lehrach, & Herwig, 2009). In this analysis, all included proteomic analytes (*n* = 171) were used as the background set.

First, the association of each identified cluster with current MDD was assessed. These results were used as a criterion for selecting cluster(s) to investigate these proteins in more detail. Linear models were constructed with each of the cluster eigenproteins as dependent variable and MDD status (3 level factor: control (reference), remitted MDD and current MDD) as an independent variable. We restricted our attention to the differences between current MDD subjects and controls, as previous research showed that proteomic differences are largest for subjects with a current – and not remitted – state of depression (Bot et al., 2015). Next, the clusters were included in a symptom-level analysis, where the relationship with the individual items of the IDS-SR_30_ was explored. Linear models were constructed with each of the cluster eigenproteins as a dependent variable and the IDs items (a four-level factor: (1) controls, (2) remitted MDD, (3) current MDD with low symptom endorsement and (4) current MDD with high symptom endorsement) as an independent variable. From these models, we performed tests comparing the controls *v.* the current MDD group with high symptom endorsement. The obtained *p* values were adjusted for multiple testing using the Bonferroni correction, where the *p* values are multiplied by the number of tests considered. Thus, for the analysis of current MDD with high symptom endorsement *v.* control the *p* values are multiplied by the number of identified clusters, and for the symptom-level analysis by the number of symptoms multiplied by the number of clusters that were significantly associated with current MDD in the main analysis. A significance level of 5% was used.

All linear models were adjusted for batch, research site, sex, age, and years of education. To investigate the effect of other health and lifestyle factors, the models were additionally adjusted for the number of self-reported chronic diseases, smoking status, alcohol consumption, and physical activity, in a separate analysis. The effect of BMI was studied by an additional correction to the health and lifestyle adjusted model.

Finally, we performed a stability analysis on the selected clusters by repeating the network analysis (1000 iterations) on the protein data of resampled sets of the original sample. For every iteration, the original sample was divided into two random subsets of size 811 and 810, and WGCNA was performed on each subset. For each network, the cluster that has the most overlap with the (original) current MDD-associated cluster was studied. Furthermore, the number of times each of the 171 proteins was contained in this cluster was reported.

## Results

### Sample characteristics

The participants (*N* = 1621 subjects) included in the analysis are characterized in Table 1. As compared to controls, subjects with current MDD (712 cases) were more likely to be female, had higher depression severity, poorer lifestyle profiles – i.e. higher BMI, more smoking, higher alcohol intake, more chronic diseases and lower physical activity – and were less educated.

**Table 1.** Sample characteristics.

	Current MDD		Remitted MDD		Healthy control		*p* value
	*n* = 712		*n* = 483		*n* = 426		
Female (*n*, %)	477	(67.0)	341	(70.6)	258	(60.6)	5.42 × 10^−3^
Age, years (mean, s.d.)	41.4	(12.3)	43.3	(12.8)	38.9	(14.8)	1.74 × 10^−5^
Education, years (mean, s.d.)	11.6	(3.2)	12.3	(3.2)	12.7	(3.1)	4.28 × 10^−9^
Body mass index, kg/m^2^ (mean, s.d.)	26	(5.5)	25.8	(4.9)	24.8	(4.6)	3.34 × 10^−4^
Chronic diseases (mean nr, s.d.)	0.7	(0.9)	0.6	(0.9)	0.4	(0.7)	2.36 × 10^−8^
Physical activity, 1000 met/day (mean, s.d.)	3.36	(3.06)	3.99	(3.23)	3.96	(3.29)	1.76 × 10^−5^
Current smoker (*n*, %)	295	(41.4)	181	(37.5)	120	(28.2)	3.88 × 10^−5^
Alcohol intake (mean nr drinks, s.d.)	5	(5.5)	4.9	(4.4)	4.7	(3.6)	3.45 × 10^−2^
Depression severity, IDS score (mean, s.d.)	31.9	(12.1)	17.6	(10.1)	8.3	(7.2)	2.20 × 10^−16^

*p* values are from a chi-square test for categorical variables or the Kruskal–Wallis test for continuous variables.

### Proteomic clusters

We identified six clusters of correlated proteins, using WGCNA (online Supplementary Table S2). For each cluster, we assessed the pathways that were enriched, using the 171 included proteins as reference (FDR<5%, online Supplementary Table S3). Cluster 1 contained 24 proteins and was enriched with 160 pathways involved in cytokine pathways, cell migration (e.g. chemotaxis), cell response and cell signaling. Cluster 2 (21 proteins) was enriched with 12 pathways, mainly pathways of the innate immune system and metabolic processes. The third cluster was enriched with 26 pathways involved in (myeloid) leukocyte immune response. Cluster 4 (10 proteins) was enriched with pathways of various metabolic processes (175 pathways) mainly driven by five apolipoproteins (ApoA1, ApoA2, ApoC1, ApoC3, ApoE). Cluster 5 consisted of seven proteins and was enriched with 17 multiple metabolic pathways. Lastly, cluster 6 (six proteins) was enriched with adaptive immune system pathways, especially T-cell mediated immunity (32 pathways). More than half of the proteins (90 of the 171) were not included in any cluster, meaning that these were not strongly connected with other groups of analytes that met the minimal cluster size of five.

### Clusters *v.* MDD

**Fig. 1.**
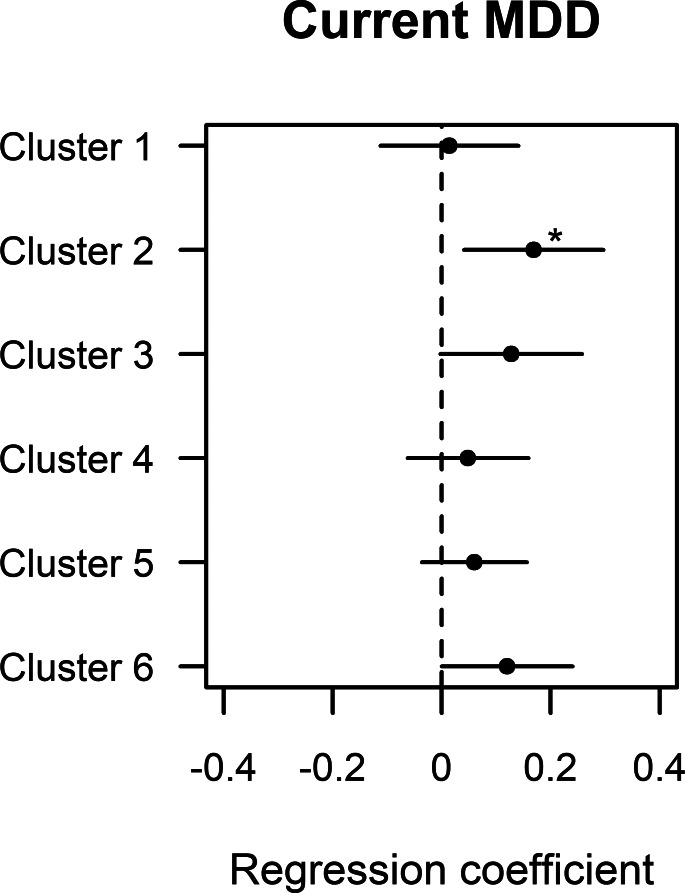
Unadjusted difference in the six proteomic clusters between current MDD subjects and healthy controls. All models were adjusted for batch, research center, age, sex and level of education. * Bonferroni adjusted *p* < 0.05.

Only one of the identified clusters (cluster 2) was significantly associated with current MDD in a model including batch, research site, sex, age and years of education (*p* = 9.62E-03, Fig. 1, online Supplementary Table S4), although the Bonferroni corrected *p*-value only reached borderline significance (adj. *p* = 0.057). The other clusters were not significantly associated with current MDD (adj. *p* > 0.3). We, therefore, chose to only focus on the cluster with the strongest association with current MDD in subsequent analyses.

Cluster 2 contained 21 proteomic analytes involved in cell communication and signal transduction (leptin, insulin-like growth factor binding protein (IGFBP)-1, IGFBP2, fatty acid-binding protein-adipocyte (FABPa), insulin, C peptide, vitronectin, fetuin A, urokinase plasminogen activator (UPA), vitamin K-dependent protein S), immune response (CRP, complement C3, haptoglobin), metabolism (adiponectin, glutathione S-transferase (GSTa), phosphoserine aminotransferase (PSAT), serum amyloid *p* component (SAP), tissue plasminogen activator (TPA), cathepsin D, prostasin) and lipid transport (apolipoprotein B). Moreover, many of these proteins play a role in inflammation or energy balance. The correlations between the proteins in cluster 2 showed that some proteins (adiponectin, IGFBP1, IGFBP2 and UPA) were negatively correlated with respect to the other analytes of the cluster (online Supplementary Fig. S1). The average absolute correlation between the 21 proteins in the cluster was 0.265. In order to quantify the relevance of each individual protein on cluster 2, we report the correlation between the proteins and cluster 2 eigenprotein (Table 2). SAP, complement C3, C peptide and insulin had the (absolute) highest correlation, indicating that these proteins play an important role in the cluster. There were four proteins that had a negative correlation with the cluster (adiponectin, IGFBP1, IGFBP2 and UPA), which is in line with the intercorrelations between the analytes within the cluster.

**Table 2.** Module membership of the proteomic analytes in cluster 2, defined as the correlation between the levels of each protein and the eigenprotein of the cluster, and the corresponding *p* values.

Analyte	Full name	Module membership	P value
APN	Adiponectin	−0.463	9.33 × 10^−^^87^
ApoB	Apolipoprotein B	0.392	1.03 × 10^−^^60^
Cpep	C-peptide	0.718	1.62 × 10^−^^257^
CRP	C-reactive protein	0.562	1.02 × 10^−^^135^
CathD	Cathepsin D	0.526	8.62 × 10^−^^116^
C3	Complement C3	0.726	2.97 × 10^−^^265^
FABPa	Fatty acid-binding protein, adipocyte	0.554	6.86 × 10^−^^131^
FetA	Fetuin A	0.350	4.69 × 10^−^^48^
GSTa	Glutathione S-transferase alpha	0.608	1.07 × 10^−^^164^
HP	Haptoglobin	0.455	9.82 × 10^−^^84^
INS	Insulin	0.707	1.43 × 10^−^^245^
IGFBP1	Insulin-like growth factor-binding protein 1	−0.461	2.78 × 10^−^^86^
IGFBP2	Insulin-like growth factor-binding protein 2	−0.512	4.34 × 10^−^^109^
Leptin	Leptin	0.516	3.31 × 10^−^^111^
PSAT	Phosphoserine aminotransferase	0.562	7.47 × 10^−^^136^
Prost	Prostasin	0.522	9.83 × 10^−^^114^
SAP	Serum amyloid p-component	0.735	1.11 × 10^−^^275^
TPA	Tissue type plasminogen activator	0.630	4.49 × 10^−^^180^
UPA	Urokinase-type plasminogen Activator	−0.429	9.46 × 10^−^^74^
VKDPS	Vitamin K-dependent protein S	0.573	2.50 × 10^−^^142^
VN	Vitronectin	0.480	3.05 × 10^−^^94^

### Clusters *v.* depressive symptoms

**Fig. 2.**
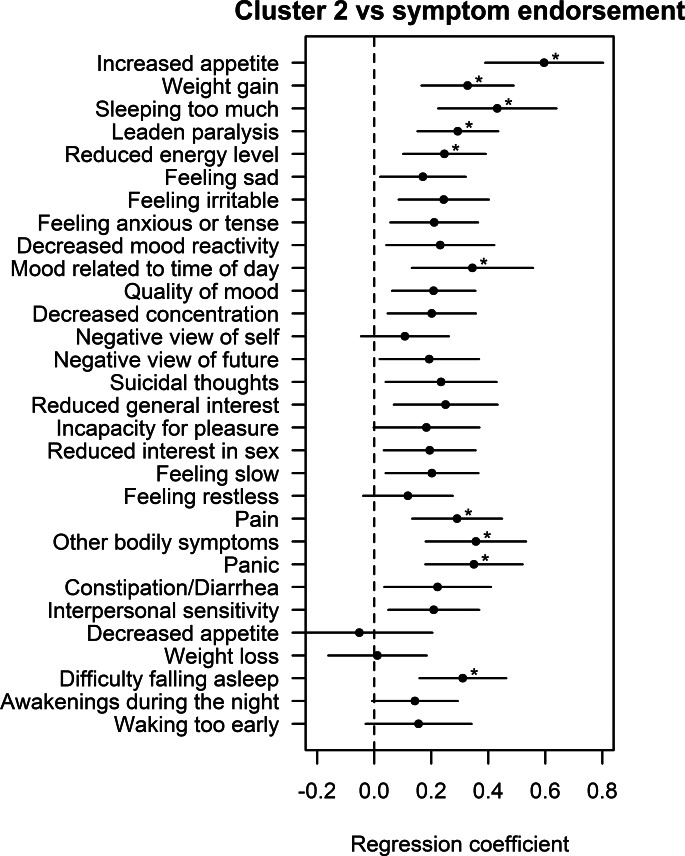
Unadjusted difference in the cluster between MDD patients with a high endorsement of specific depressive symptoms and healthy controls. All models were adjusted for batch, research center, age, sex and level of education. * Bonferroni adjusted *p* < 0.05.

Next, the association with each of the 28 selected symptoms from the IDS was investigated, while correcting for batch, research site, sex, age and years of education. As compared to controls, current MDD subjects with a high endorsement of the following symptoms had a significantly higher level of cluster 2: increased appetite (*p* = 3.79–08), hypersomnia (*p* = 4.79 × 10^−5^), leaden paralysis (*p* = 5.34 × 10^−5^), panic (*p* = 6.14 × 10^−5^), difficulty falling asleep (*p* = 6.75 × 10^−5^), weight gain (*p* = 7.56 × 10^−5^), other bodily symptoms (*p* = 7.74 × 10^−5^), pain (*p* = 3.12 × 10^−4^), reduced energy level (*p* = 8.72 × 10^−4^), and mood related to time of day (*p* = 1.55 × 10^−3^) (Fig. 2 and online Supplementary Table S5). When comparing the expression of the cluster between current MDD subjects with low symptom endorsement of each symptom and healthy controls, there were no significant associations (online Supplementary Fig. S2 and Table S6). As post-hoc analysis, we repeated the symptom-level analysis for the remaining five clusters (online Supplementary Table S7). There were no significant associations between the five clusters and any of the symptoms, after Bonferroni correction.

### Stability of the cluster

**Fig. 3.**
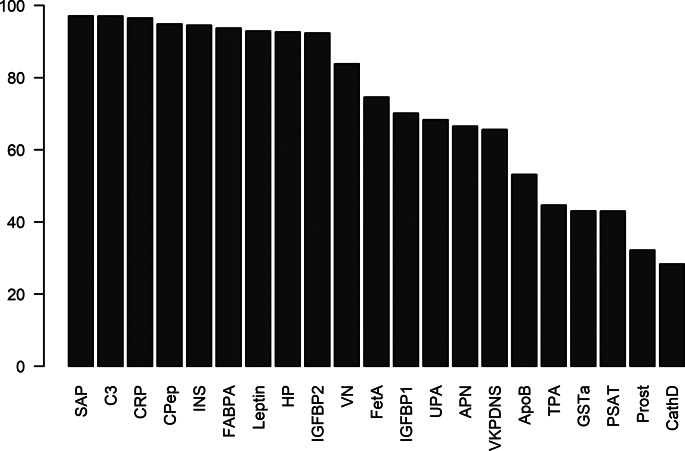
Percentage of iterations (of 2000) that the proteins were contained in the cluster that had most overlap with the immune-metabolic cluster. Only proteins that were contained in the original cluster are displayed.

The stability of cluster 2 was investigated by repeating the WGCNA network analysis on the protein data of resampled sets of the original sample. For 1000 iterations, we divided the original sample into two random subsets and WGCNA was performed on both subsamples, resulting in 2000 sets of clusters. For each set, the cluster that had the most overlap with cluster 2 was investigated. The average size of this cluster was 18.8 (s.d. = 4.7) and the mean overlap with cluster 2 was 15.2 (s.d. = 3.9), out of 21 proteins. This shows that the identified cluster is highly stable and not dependent on a specific subsample. The number of iterations each protein was contained in the most-overlapping cluster was used to identify the proteins that are important and stable in the MDD-associated cluster. Especially SAP, C3, CRP, Cpep, insulin, FABPa, leptin, HP and IGFBP2 appeared to play an important role, since these proteins were contained in the largest overlapping cluster in more than 90% of the iterations. Also VN, fetuin A, IGFBP1, UPA, APN, VKDPS and ApoB were contained in this cluster in more than 50% of the iterations (Fig. 3, online Supplementary Table S8). TPA, GSTa, prostasin and cathepsin D appeared to be the least stable proteins of cluster 2.

### Health and lifestyle factors

To investigate the influence of health and lifestyle factors on the relationship between the clusters and MDD status, the following covariates were added to the models: the number of self-reported chronic diseases, alcohol consumption, smoking and physical activity. None of the clusters was significantly associated with current MDD when adding these variables (online Supplementary Table S4). However, in the symptom-level analyses, the significant relation between the following symptoms and cluster 2 remained (online Supplementary Table S5): increased appetite (*p* = 8.39 × 10^−7^), weight gain (*p* = 9.70 × 10^−4^), panic (*p* = 1.11 × 10^−3^) and hypersomnia (*p* = 1.50 × 10^−3^). The additional correction for BMI in the model that investigated the relationship between MDD status and the clusters led to a large change in the corresponding regression coefficients (online Supplementary Table S4). In the symptom level analysis, none of the results remained significant after correcting for BMI (online Supplementary Table S5).

## Discussion

By applying WGCNA on proteomic assay data, we identified six clusters of correlated proteins, from which one cluster was nominally associated with current MDD. This cluster contained 21 proteins - including leptin, insulin and CRP - mainly involved in inflammation and metabolism. The cluster was associated with ten depressive symptoms, among which five energy-related symptoms that are thought to be closely linked to an immuno-metabolic form of depression (IMD), and the strongest relations were with the energy-related symptoms increased appetite and hypersomnia. CRP, leptin and insulin are known to be among the key biomarkers altered in MDD, and even more pronouncedly in patients with IMD (Milaneschi et al., 2020). Here we showed that not only these three proteins, but a larger correlated protein network including also IGFBP2, FABPa and C3, is associated with symptoms linked to IMD. These results support the idea that a network of inflammatory and metabolic proteins represents network-level mechanisms that are related to the full spectrum of energy-related IMD symptoms.

In a previous study using the same proteomic immunoassay data measured in NESDA, we evaluated changes in individual protein levels in two data-driven MDD subtypes that were most distinguished by the direction of appetite and weight change during a depressive episode (Lamers et al., 2016). In contrast with the present study, endorsed symptoms were first clustered into subtypes and thereafter the associations of these subtypes with individual proteins was investigated. From the 23 proteomic markers associated with the atypical depression subtype characterized by increased appetite and weight in this prior work, we found that 16 of these were included in a cluster that differed between current MDD subjects and controls. Five of the individual symptoms associated with the cluster are also linked to the atypical depression subtypes studied in Lamers et al. (2016). Thus, these results are similar to the findings of the present study, despite the different approaches used. This suggests consistent relations between the proteins (cluster) and atypical, energy-related symptoms, irrespective of the analytical approach. In other NESDA research, latent class analysis (LCA) was used to cluster patients based on 36 biomarkers (not including the proteomic assay used for the current study) (Beijers et al., 2019). The three identified classes were partially determined by the biomarkers CRP and leptin, however, the classes were not characterized by differences in depressive symptom endorsement, showing that the classes found by Beijers do not overlap with energy-related symptoms (profiles).

It should be noted that the identified cluster is not exclusively associated with symptoms linked to IMD. These other symptoms were mood in relation to the time of day, panic, pain, other bodily symptoms, and difficulty falling asleep. Their association could be explained by the possibility that the cluster represents more than the biological mechanism underlying IMD. For instance, the latter three symptoms could represent underlying somatic disease that is linked to cluster 2 proteins. Alternatively, this finding could indicate that the biological mechanism reflected by the cluster is linked to a broader range of symptoms than only IMD symptoms.

It is known that CRP, insulin and leptin interact in metabolism pathways and it was proposed that their joint measurement may be an improved marker for cardiovascular disease, obesity and depression (Hribal, Fiorentino, & Sesti, 2014; Milaneschi, Simmons, van Rossum, & Penninx, 2019; Sudhakar, Silambanan, Chandran, Prabhakaran, & Ramakrishnan, 2018). We showed that CRP, insulin and leptin are amongst the most stable proteins in the network, but also SAP, C3, Cpep, FABPa, HP and IGFBP2 appear to play an important role based on the cluster-stability analysis. Some of these proteins are known to interact: SAP, CRP, and HP are acute phase (inflammatory) proteins, CRP and SAP have 51% sequence homology, and C-peptide (Cpep) is crucial in the insulin biosynthesis pathway. Fatty acid-binding protein adipocyte (FABPa) has been associated with lipid metabolism disorders, diabetes and obesity and interacts with leptin (Gan, Liu, Cao, Zhang, & Sun, 2015). Complement component 3 (C3) is a protein of the immune system, with a central role in the complement system. C3 expression is increased in the prefrontal cortex (PFC) of depressed subjects, and selective overexpression of C3 in PFC of mice was sufficient to cause depressive-like behavior in mice (Crider et al., 2018). Also, leptin levels were reduced in mice in C3(-/-) mice, indicating the interaction between leptin and C3 and the role of C3 in energy balance (Murray, Havel, Sniderman, & Cianflone, 2000). Insulin-like growth factor-binding protein 2 (IGFBP2) is linked to insulin sensitivity (Yau et al., 2018). Although pairwise interactions from many of the 23 proteins in the immuno-metabolic cluster are known, we show that they may jointly contribute to a network-level mechanism, which is dysregulated in patients with IMD.

In other studies using single marker protein measures in NESDA, higher CRP was associated with increased appetite (Lamers, Milaneschi, de Jonge, Giltay, & Penninx, 2018) and higher leptin with mood dependent on the time of day, decreased appetite, increased appetite, weight gain, reduced energy level, pains, other bodily symptoms and leaden paralysis (Milaneschi, Lamers, Bot, Drent, & Penninx, 2017). Compared to these single analyte analyses, the cluster we identified was associated with more atypical, energy-related symptoms, which may indicate that a single protein marker does not capture complete symptom profiles. However, it should be noted that in this previous research, the associations between individual symptoms and serum levels of CRP and leptin were only investigated within the currently depressed sample. In the present study, the symptom-level results were obtained from the analysis that compared current MDD patients with high symptom endorsement to healthy controls. We also evaluated differences in cluster 2 between cases with low symptom endorsement and healthy controls, and none of the symptoms differed significantly between the two groups. Therefore, we hypothesize that the high endorsement of specific symptoms drives the associations, rather than the presence of MDD in general.

In the present analyses, correction for various health and lifestyle covariates reduced the effect size of the associations between the protein cluster and individual symptoms. As in previous research, this suggests that a substantial proportion of the protein-symptoms associations is explained by health and lifestyle, which may be an indicating that lifestyle adjustments could reduce the deviance of protein levels in patients with depression. In additional analyses further correcting for BMI, all protein-symptom associations were substantially reduced and no longer significant. However, evidence suggests that MDD shares genetic risk factors with both inflammation and BMI (Milaneschi, Lamers, Peyrot, et al., 2017; Van Den Broek et al., 2018; Wray et al., 2018), and the position of BMI in the pathways that link inflammation and depression is complex. Therefore, adjusting for BMI may lead to overadjustment in the association between inflammatory markers and MDD (Lasserre et al., 2014; Luppino et al., 2010). Antidepressant use was not included in the analyses as a potential confounder because earlier NESDA analyses using the same proteome platform found that antidepressant use did not play a confounding role in the relationship between MDD and proteomics (Lamers et al., 2016). Despite indications that robust cluster structures are absent in a subset of the proteomic dataset that was used in the present paper (Beijers et al., 2020), our analyses showed that most of the proteins from cluster 2 are strongly correlated and together form a stable cluster. Moreover, our results converge with previous studies on symptoms and inflammatory/metabolic markers (Lamers et al., 2018; Milaneschi, Lamers, Bot, et al., 2017).

The present finding may have several implications. The identified cluster could contribute to the stratification of patients by immuno-metabolic dysregulation in combination with the endorsement of specific IMD symptom patterns. There is evidence that there is a specific subgroup of MDD patients with an underlying immune dysregulation that does not respond to antidepressant treatment (Kopschina Feltes et al., 2017). It is hypothesized that this subgroup could benefit from anti-inflammatory treatment. The presence of the atypical, energy-related symptom patterns linked to IMD associated with the identified cluster suggest immunological and metabolic alterations, and might be used as stratification for anti-inflammatory treatment (Milaneschi et al., 2020). However, more research is required to investigate the relationship between the cluster and the depressive symptoms in more detail, and the usability of measuring a protein cluster in routine practice. The proteomic platform used here measured a limited amount of proteins: the identified cluster may be part of a larger protein network not captured by this platform. Which, and how many markers of this network are optimal for representing this network, in order to stratify patients, has to be studied further.

Strengths of this study include the relatively large sample size with subjects recruited from the general population, primary care and specialized mental health care. Second, this study has investigated the association of the clusters with MDD status as well as specific symptoms. This provides insight into the relation of the clusters with depression in general, as well as with more homogenous symptom patterns. Third, by using clusters we were able to evaluate the combined effect of many correlated proteins on several depressive symptoms simultaneously. Analyzing such a large number of proteins separately would require many more tests, which would lead to a substantial loss of power due to correction for multiple testing.

Limitations of this study include the fact that some analytes could not be investigated due to many missing values. Besides the information loss, the exclusion of analytes can cause alterations in the constructed network (Pei, Chen, & Zhang, 2017). Ideally for WGCNA, the missing values of each analyte would be replaced according to some imputation method. However, these methods become inaccurate with an increasing number of missing values (Lazar, Gatto, Ferro, Bruley, & Burger, 2016). Therefore, we have excluded analytes with >30% missing values. Finally, our proteomic platform is covering a rather selective part of the proteome. Consequently, there may be (many) more proteomic pathways related to MDD that were not captured through our methods.

In conclusion, by using WGCNA to cluster proteomic analytes, we were able to identify a cluster – consisting of proteins involved in inflammation and metabolism – that is linked to MDD, and more specifically to MDD patients with specific symptoms that are thought to be important characteristics of IMD (Milaneschi et al., 2020). Our findings suggest that CRP, leptin and insulin are part of a larger network of proteins that plays an important role in the biological processes underlying IMD. This cluster of biomarkers in conjunction with an endorsement of behavioral IMD symptoms may provide to a more suitable tool for patient stratification compared to single biomarkers.

## Supplementary material

For supplementary material accompanying this paper visit https://doi.org/10.1017/S0033291721004888. click here to view supplementary material
