# Supplementary figures and images for: Dissection of depression heterogeneity using proteomic clusters
Source: Psychol Med. 2022 Jan 18;53(7):2904–12. doi: 10.1017/S0033291721004888 (PMC10235664; doi:10.1017/S0033291721004888)

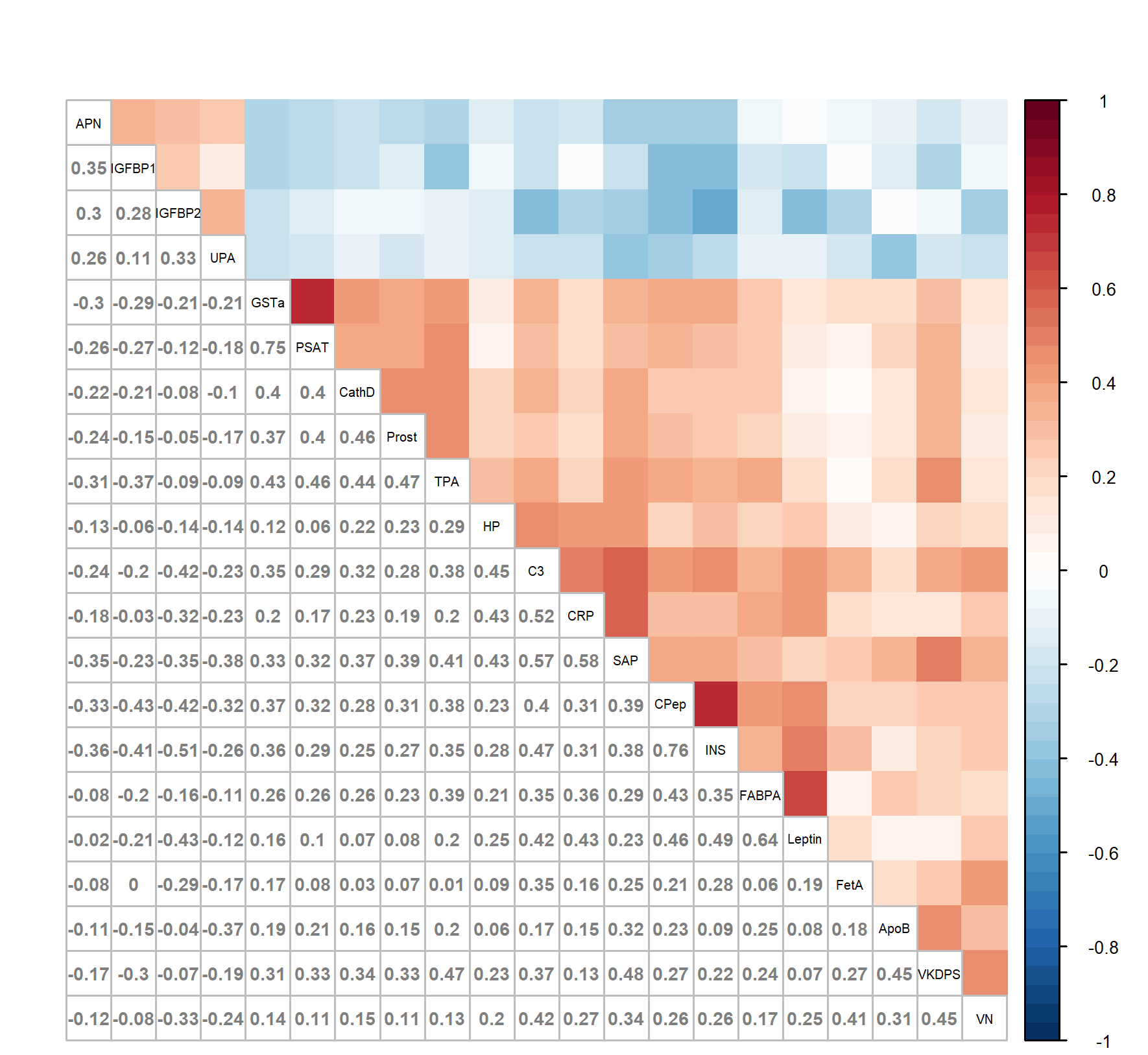

Supplement: Supplementary file 1 [file S0033291721004888sup.zip › S0033291721004888sup001.tiff]
